# Impact of value similarity on social trust in medical students: a cross-sectional web survey

**DOI:** 10.1186/s12909-023-04493-w

**Published:** 2023-07-24

**Authors:** Satoshi Kondo, Shuhei Ichikawa, Masashi Izumiya, Masato Eto

**Affiliations:** 1 Department of Medical Education Studies, International Research Center for Medical Education, Graduate School of Medicine, The University of Tokyo, Bunkyo-ku, Tokyo, Japan; 2 Department of Medical Education, Graduate School of Medicine, University of Toyama, Toyama, Japan; 3 Department of General Medicine, Mie University Graduate School of Medicine, Mie, Japan; 4 Clinical Simulation Center, The University of Tokyo Hospital, Tokyo, Japan; 5 Department of Gastroenterology, The University of Tokyo Hospital, Tokyo, Japan

**Keywords:** Social trust, Student doctor, Certification system of medical education, Salient value similarity model, Value similarity, Risk reduction, Medical communication, Acceptance of medical practice

## Abstract

**Background:**

Social trust in medical students is trust in the cluster of medical students and not individual medical students. Social trust in medical students seems critical in clinical practice since citizens often face unknown medical students for the first time. However, most previous research has focused on interpersonal trust in particular medical professions, and social trust in medical students has not been addressed sufficiently. In social science, the Salient Value Similarity model has demonstrated that the value similarity between professionals and citizens is associated with social trust. This research aimed to explore the relationship between social trust in medical students and the perception of value similarity. This study also aimed to determine whether the information of medical students strengthens social trust in them.

**Methods:**

We conducted a cross-sectional study to investigate how the perception of value similarity affects social trust. The participants answered the social trust questionnaires before and after reading a brief summary of the medical education curriculum and certification via the internet in Japan. The model structure of social trust in medical students, including the perception of value similarity, was investigated using SEM. A paired *t*-test was used to examine the effect of informing citizens about the knowledge, skills, and professionalism requirements of students attending medical school on social trust by reading the brief summary.

**Results:**

The study included 658 participants, who all answered a web questionnaire. Social trust in medical students was associated with the perception of ability and value similarity. Social trust in medical students, the perception of ability, and value similarity were improved by information about medical students.

**Conclusions:**

The perception of ability and value similarity seem to affect social trust in medical students. Information on medical education regarding the knowledge, skills, and professionalism of medical students may improve social trust in these students. Further research is required to sophisticate the model of social trust in medical students by exploring social trust in the medical students’ supervisors in clinical settings.

**Supplementary Information:**

The online version contains supplementary material available at 10.1186/s12909-023-04493-w.

## Background

What facilitates the trust of citizens in unknown medical students? The trust of patients in physicians is the core concept of the patient–doctor relationship [1, 2] and is essential to medical practice [3]. Furthermore, trust is becoming even more essential in medical education settings, where supervisors need to provide medical students with clinical-procedure opportunities [4].

Trust in medicine is defined as the citizens’ expectations of medical professionals who perform for citizens competently, responsibly, and act by placing patients first [1]. Trust comprises two aspects, social trust and interpersonal trust [5, 6]. Social trust in physicians is the trust of citizens in physicians in general. Social trust is the trust in the cluster of individuals who manage safety [7], such as physicians. Social trust is more conceptual than interpersonal trust [8]. Interpersonal trust in physicians addresses a particular physician who has a relationship with the person. Social trust is the foundation of interpersonal trust and has a complementary function to interpersonal trust [6, 8–10]. However, trust in physicians, which has remained high, could be impaired by increasing information to healthcare professionals because of improved transparency and changes in the healthcare system [11, 12]. The loss of social trust does not pertain to individual physicians; rather, it pertains to all physicians in general [6, 13, 14]. In other words, social trust in doctors as a whole is diminishing. In fact, social trust in physicians is lower than interpersonal trust in physicians [10].

Social trust in medical students seems to be more critical than interpersonal trust in the context of medical education. First, most patients do not have a sufficient relationship with and information about individual students in a clinical setting. Therefore, patients are required to use social trust in medical students for trusting them. Similarly, when medical education researchers consider the patients’ trust in medical students, they need to focus on social trust in medical students. Furthermore, medical students have a marginal position between laypersons and professionals. Therefore, the study of social trust in medical students cannot be substituted by the study of social trust in physicians. In other words, the study of social trust in medical students is essential in medical education research and social psychology research.

Trust within the healthcare system has been a focus of research in the past 20–30 years. In the context of medical education, the research on trust has encountered three challenges. First, the focus on social trust in medicine is developing. Most previous studies have assessed the trust in physicians among patients [2], which was not sufficient to evaluate the social trust in physicians by citizens. Second, there is a lack of social trust research in medical students. Previous research addressed the trust in doctors, nurses, pharmacists, researchers, health systems, and insurers, disregarding medical students [4, 15]. Third, approaches to the concept of trust are in development [5]. The precise conceptual framework is vital to evaluate trust in medical practice [16]. Subsequently, the introduction of a conceptual framework is required.

Which conceptual model should be used in medical education? Some models have been developed on trust in physicians [2, 6, 15]. However, few statistically evaluated models of social trust in medical students and healthcare professionals have been developed [5]. In this study, we introduce value similarity to explore social trust in medical students by selecting the Salient Value Similarity (SVS) model. The SVS model is used in risk perception research, and this theory contends that trust receives a contribution from the following constructs (factors): value similarity and ability [7, 17]. In contrast, the traditional trust theory is affected by ability and motivation. The SVS model is characterized by the fact that trust is affected by each citizen’s perception that their values are similar to those of professionals. In addition, citizens weigh the risks and benefits based on trust [7, 17]. Trusting doctors or medical students to perform medical procedures is truly a risk perception based on trust [4]. The perception of having a patient-centered value may improve trust in physicians, which implies that value similarity between the patient and the physician will affect trust [12, 18, 19]. Moreover, the adaptation of the SVS model seems to be helpful for medical education, to consider social trust in medical students. However, the SVS model has not been introduced in medical education research to seek and develop social trust in medical students. The model structure of previous studies was adopted from the SVS model [7, 17] regarding the perception of trust and risk reduction, including value similarity, ability, and motivation [20–24]. Social science researchers have adapted and verified this model in the US [21] and Japan [23]. In this study, we assumed that the model structure comprised five factors, i.e., citizens’ perception of trust, value similarity, ability, motivation, and risk reduction.

The primary aim of this study was to explore the impact of value similarity perceptions by citizens on social trust in medical students. The secondary aim was to assess the effects on value similarity, ability, motivation, and trust of reading a brief summary of the medical education system and the certification of medical students.

The evaluation of the impact of value similarity on social trust in medical students may provide new knowledge on how to improve social trust in medical students and accept medical practice by medical students. If the impact of value-sharing on trust could be identified in this study, it is possible that the application of the SVS model can be expanded to explain interpersonal trust in medical students. Furthermore, exploring this hypothesis will provide information on the importance of public communication about the education system of medical schools and the certification of medical students.

Our hypotheses were as follows:

1. Social trust in medical students is associated with citizens’ perception of value similarity about medical students.
2. Informing citizens about the certification of medical students (student doctors) and the education system in medical schools strengthens social trust in medical students through a perception of their ability.

To test our hypotheses, the participants who were recruited into the study completed a web survey about social trust in medical students using an SVS model-based modified questionnaire for evaluating social trust in medical students. The reliability of the original questionnaire was verified and validated in Japan [20, 24]. In addition, the citizens were informed by a brief summary about the medical education system and certification, followed by a repeat questionnaire to assess social trust in medical students. All participants filled out the questionnaire twice. Initially, the participants filled out the questionnaire without knowledge about medical students, and the assessment performed at this time represented social trust without this knowledge. Thereafter, the same participants filled out the questionnaire again after reading a brief summary about medical students. We designed this questionnaire survey as a simulation of the distribution of an explanatory document to a patient and requesting blood sampling in a clinical context after reading the brief summary to an individual who as unfamiliar with medical students. Blood sampling was chosen because this invasive medical procedure is imaginable and familiar to citizens in Japan.

We believe this study will contribute to scientific development in the following two aspects: First, this study will provide insight into professionals with a boundary position. Previous trust research in social psychology has primarily examined trust in professionals. The results of this study could be extended to understanding trust not only in the medical professions but also in students and novice professionals in a wide range of fields. Second, this study will clarify the relationship between acceptance for blood sampling and social trust by applying the SVS model. Prior research has only explored trust and the factors involved. The focus on the relationship between the citizen’s choice and social trust distinguishes this study from previous ones. In these respects, the results appear to be both novel and relevant.

## Methods

### Research design

A cross-sectional study using a web-based questionnaire was conducted to identify the factors that have a more critical effect on the trust of citizens in medical students at each of the two-time points, before and after reading the brief summary.

### Setting and data collection

This survey was conducted from November 19 to November 22, 2021, and was distributed through an internet research company.

### Participants and recruitment

To recruit the general population as our participants, we employed an internet survey company. The inclusion criteria were community-dwelling people aged 20 years or more who lived in the following prefectures: Tokyo, Kanagawa, Saitama, and Chiba. This area encompasses 30 million people, which is more than 25% of Japan’s population, and represents the Tokyo metropolitan area. Stratified random sampling according to sex, age group, and living prefecture was used to assure the representativeness of the samples. The people who did not accept to participate were excluded from this study. The rates of strata were set as following: gender (female:male = 1:1, option “other” were accepted), age group (10–19:20–29:30–39:40–49:50–59:≥60 = 1:1:1:1:1), and living prefecture (Tokyo:Saitama:Chiba:Kanagawa = 2:1:1:1).

Invitations via the internet were sent to registered monitors in an internet survey company, and we allocated individuals who accepted the invitations as participants. We asked the research company to ensure no missing data and to design the web survey. The participants received points that could be exchanged for cash. We decided to recruit individuals who were unfamiliar with hospitals and medical students through the internet research company, rather than recruiting participants living near our medical schools and university hospitals. To investigate social trust in medical students, we needed to recruit persons who had not experienced medical practice by students. Of course, every citizen in Japan has visited a hospital or engaged with a medical student, to a certain degree. However, we expected that recruiting participants through a research company would minimize the familiarity with hospitals and medical students compared with recruiting participants at university hospitals. Furthermore, the following factors were identified in social science as affecting the decision to evaluate the risk and benefit: citizens’ features, professional or not; cultural values; knowledge; and gender [7]. Therefore, we attempted to increase the diversity of the participants by utilizing subject recruitment through a research firm.

### Questionnaire/scale

**Fig. 1:**
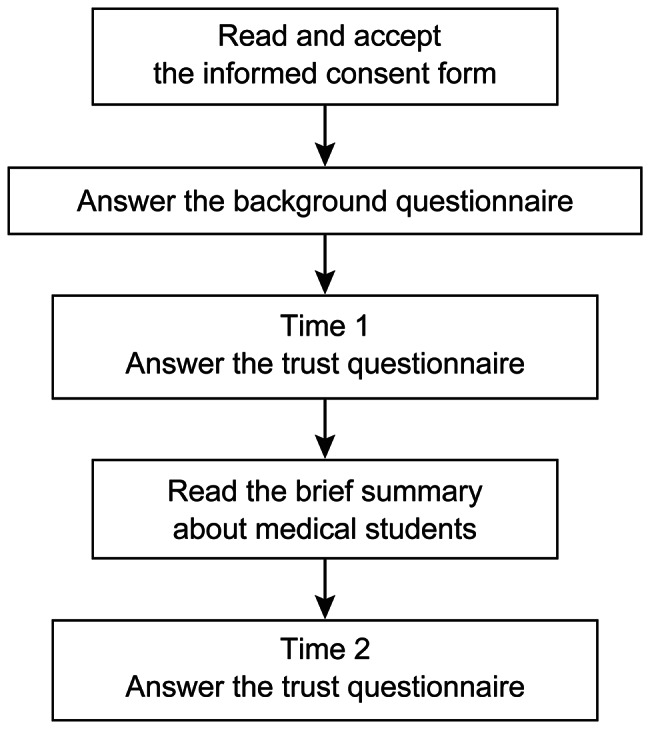
Flow diagram of the experience of the participants in the current research

The participants’ experience in this research is shown in Fig. 1. The questionnaires consisted of five sections. First, the informed consent document was presented, and those who gave consent to participate filled in the questionnaires. Second, a background questionnaire was administered that contained the following items: gender (male, female, non-response), age, prefecture, job (nurse, doctor, other), the experience of teaching medical students, and the experience of hospitalization or regular visits to clinics/hospitals. Third, a trust questionnaire (Additional file 1) and a question regarding the acceptance for blood sampling were administered. The trust questionnaire included 15 items. The present study’s questionnaire was based on a questionnaire that was previously tested regarding its reliability and validity [20, 24]. The questionnaire consisted of five factors, the perception of trust, value similarity, ability, motivation, and risk reduction in medical students. Trust was measured by questions No. 1–3. Value similarity was measured by questions No. 4–6. Ability was measured by questions No. 7–9. Motivation was measured by questions No. 10–12. Risk reduction was measured by questions No. 13–15. We added a question to evaluate the acceptance for blood sampling as the surrogate indicator of the action of citizens in the clinical context. Acceptance for blood sampling was measured by question No. 16. These 16 items were presented on one page. Fourth, a brief summary (Additional file 2) was used to present the medical education curriculum, the certification system of student doctors, and the medical students’ learning experience in Japan. The brief summary explains that medical students must fill in the requirements for knowledge, skills, and professionalism. Fifth, the trust questionnaire and the question regarding the acceptance for blood sampling were administered a second time; i.e., the participants were first asked to answer the questionnaire, then asked to read the brief summary and answer the questionnaire again. We conducted the questionnaire a second time by assuming to request a blood sample after distributing the brief summary about medical students.

The trust questionnaire comprised the following five factors: trust, value similarity, ability, motivation, and risk reduction. Each factor had three items. Thus, the trust questionnaire included a total of 15 items. The trust questionnaire and the acceptance for blood sampling were rated on a 5-point Likert scale, with 5 indicating that the participant agreed very strongly and 1 indicating total disagreement. The sum of items in each factor of the trust questionnaire was used as the component score of trust. The scores on trust, value similarity, ability, motivation, and risk reduction had a range of 3–15 points, whereas the score on the acceptance for blood sampling had a range of 1–5 points.

### Statistical analyses

**Fig. 2:**
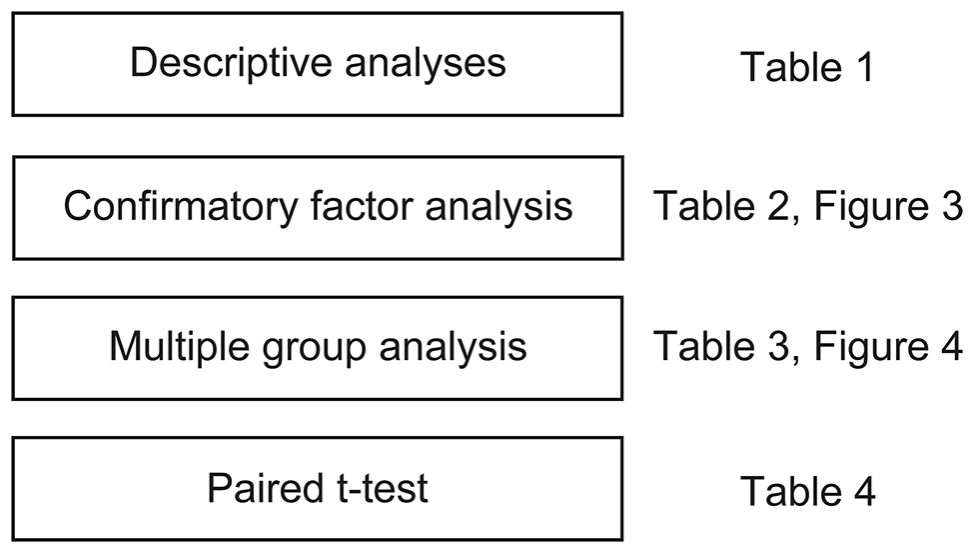
Statistical analyses performed in this research

The statistical analyses performed in this research are shown in Fig. 2.

First, descriptive analyses were conducted regarding participants’ features, including sex, age, residence, job (nurse or doctor), experiences of hospitalization, regular visits, and experiences with medical students.

Second, a confirmatory factor analysis (CFA) was conducted on the trust questionnaire administered in this study using the SVS model structure described in previous studies [7, 20, 24]. Cronbach’s alpha and McDonald’s omega were calculated to confirm the internal consistency of each factor in the trust scale [25]. After the CFA, structural equation modeling (SEM) was performed to identify the suitable model for explaining social trust in medical students.

Third, a serial multiple group analysis (MGA) with mean structure using SEM was conducted as the primary analysis. MGA enables the comparison of the model structure between strata, such as two groups or sequential measurements [26]. Therefore, we tried to examine the equivalences of the model structures using MGA before and after reading the brief summary. After this procedure, a comparison of scores before and after reading the brief summary was performed using the paired *t-*test. Through MGA, we performed a stepwise comparison of the fit indices between model structures that have fewer constraints and more constraints. The model with the most constraints is selected if fit indices do not deteriorate significantly in MGA. In case the model structures can be regarded as similar, we can compare the scores of the social trust questionnaire before and after reading the brief summary. In Model 1, the same constructs from the same observed variables were assumed, and none of the model parameters were constrained as being the same between time points (configural invariance). In Model 2, factor loadings were constrained as being the same between time points, in addition to Model 1. In Model 3, factor loadings and intercepts were constrained as being the same between time points. In Model 4, factor loadings, intercepts, and measurement errors were constrained as being the same between time points. In Model 5, factor loadings, intercepts, measurement errors, and path coefficients were constrained as being the same between time points. In Model 6, factor loadings, intercepts, measurement errors, path coefficients, and factor means were constrained as being the same between time points. If those models were rejected, the model structure between strata was regarded as being different [27]. Robust maximum likelihood estimation was used. The Satorra–Bentler chi-squared test was used to compare the fit indices of those models [28]. The comparative fit index (CFI ≥ 0.95), standardized root mean square residual (SRMR < 0.05), and root mean square error of approximation (RMSEA < 0.08) were used to confirm the model fit [29–31].

Finally, a paired *t*-test was conducted to detect changes associated with the brief summary. Significance was set at *P* < 0.05.

Statistical analyses were conducted using R 4.1.2, RStudio Build 554, and the following packages: tidyverse, readr, tableone, lavaan, lme4, semPlot, semTools, psych.

## Results

In total, 658 individuals participated in this research. Table 1 lists the characteristics of the study participants. The female and male genders were approximately proportional. The average age of the participants was generally equal to that of the Japanese population. Very few doctors and nurses participated in the study. About three-quarters of the participants did not engage with medical students.

**Table 1:** Descriptive statistic: participant characteristics

**Overall**	
N	658
Sex	
1 Male, No. (%)	306 (46.5)
2 Females, No. (%)	303 (46.0)
3 Others, No. (%)	49 (7.4)
Age	
Mean (SD), years	45.21 (15.02)
Range, years	20–88
Residence (%)	
11 Saitama Pref.	131 (19.9)
12 Chiba Pref.	129 (19.6)
13 Tokyo Pref.	266 (40.4)
14 Kanagawa Pref.	132 (20.1)
**Q1 Are you a nurse or doctor? (%)**	
1 No, No. (%)	645 (98.0)
2 Nurse, No. (%)	11 (1.7)
3 Doctor, No. (%)	2 (0.3)
If Q1 = 2 or 3 (Nurses and doctors, Total 13), then answer Q2	
**Q2 Have you ever taught medical students for medical practice (medical interviews, physical examinations, blood samplings, etc.) when you worked as a medical staff member?**	
1 Yes, No. (%)	7 (53.8)
2 No, No. (%)	6 (46.2)
**Q3 As a patient or patient’s family member, have you ever regularly visited a hospital or clinic or been hospitalized in the past?**	
1 Yes, No. (%)	419 (63.7)
2 No, No. (%)	239 (36.3)
**Q4 As a patient or a patient’s family member, when you visited a hospital or a clinic, did you ever receive medical treatment such as an interview, medical examination, or blood sampling by medical students?**	
1 Yes, No. (%)	182(27.7)
2 No, No. (%)	476 (72.3)

**Fig. 3:**
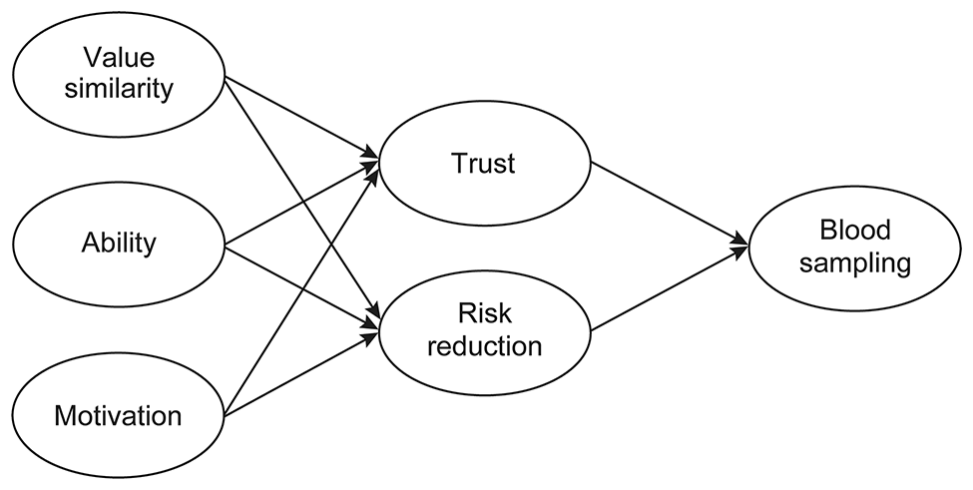
Medical Students-Social Trust model (MS-ST model) structure of the present study

Table 2 shows the results of the CFA and SEM. The CFA was conducted using the five factors reported in previous studies, and a good fit was obtained [20, 24]. Five factors comprised the perception of trust, value similarity, ability, motivation, and risk reduction in medical students. The Cronbach’s alpha coefficients for each factor in the trust scale were > 0.85, and the McDonald’s omega coefficients for each factor was > 0.85. After the CFA, the model structure of the trust scale and blood sampling was explored using SEM. Because the goodness of fit for the SVS model initially assumed was not sufficient (SRMR > 0.05, Robust RMSEA > 0.080), we prepared a modified model in which risk reduction was set to the second level, same as trust. Those models are shown in Additional file 3. The modified model showed adequate goodness of fit. The fit indices of the modified model were improved more than the SVS model initially assumed. Furthermore, those indices were within the confirmation of the model fit in this study, as shown in the Methods section (Robust CFI ≥ 0.95, SRMR < 0.05, and RMSEA < 0.08). At the next level of trust and risk reduction on the modified model, the acceptance for blood sampling was set (Medical Students-Social Trust model (MS-ST model), as shown in Fig. 3). The MS-ST model showed a good fit and was accepted. The goodness of fit indices for each model is shown in Table 2.

**Table 2:** Fit indices indicated by the confirmatory factor analysis and structural equation modeling

	Robust CFI	SRMR	Robust RMSEA	90% confidence interval—lower	90% confidence interval—upper
CFA*					
5 factor model	0.975	0.039	0.063	0.053	0.074
SEM**					
SVS model***	0.954	0.064	0.085	0.076	0.095
Modified model	0.975	0.039	0.063	0.053	0.074
MS-ST﻿ model	0.972	0.04	0.064	0.055	0.073

Robust maximum likelihood estimation was used for parameter estimation

* CFA: Confirmatory factor analysis

**SEM: Structural equation modeling

*** SVS model: Salient Value Similarity model

**Fig. 4:**
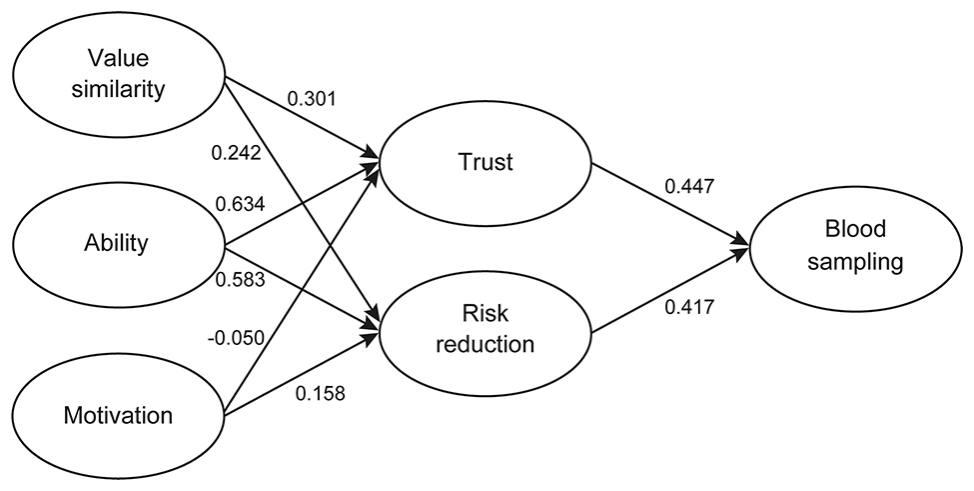
MS-ST model structure and path coefficients before reading the brief summary

Table 3 reports the result of the MGA. Model 2 was accepted, which means that the factor structures and factor loadings were equivalent before and after reading the brief summary. This allows us to compare the scores of the social trust questionnaire before and after reading the brief summary. Figure 4 depicts the path coefficients obtained by MGA in the MS-ST model before reading the brief summary of the medical education curriculum and certification of student doctors. The MS-ST model was a good fit.

**Table 3:** Fit indices indicated by multiple group analysis with mean structure

Model	BIC	Robust CFI	SRMR	Robust RMSEA	Chi square difference*	P*
Model 1	38,192	0.975	0.034	0.066	-	-
Model 2	38,132	0.975	0.035	0.064	10.518	0.3963
Model 3	38,128	0.972	0.036	0.066	69.045	< 0.001
Model 4	38,227	0.963	0.057	0.073	94.804	< 0.001
Model 5	38,188	0.962	0.069	0.072	13.314	0.1015
Model 6	38,212	0.960	0.084	0.073	64.785	< 0.001

Model 2 was selected since the fit indices in Model 3 significantly deteriorated

Robust maximum likelihood estimation was used for parameter estimation

* Differences in chi-squared between models and P-values were calculated using the Satorra?Bentler method

The brief summary contained information about the medical education curriculum and certification of student doctors.

Table 4 reports the results of the paired *t*-test. After reading the brief summary, the participants scored significantly higher on each item of perception of trust, value similarity, ability, motivation, risk reduction, and blood sampling. The significance levels of trust, value similarity, ability, risk reduction, and the acceptance for blood sampling was *P* < 0.001, and that of motivation was *P* < 0.05.

**Table 4:** Paired *t*-test stratified according to time

	Time 1 mean (SD)	Time 2 mean (SD)	Mean of the differences	*P*-value	95% confidence interval	
Total score on trust, three items	7.52 (2.46)	8.64 (2.76)	1.12	< 0.001	0.98	1.27
Total score on value similarity, three items	7.89 (2.54)	8.62 (2.66)	0.74	< 0.001	0.60	0.87
Total score on ability, three items	8.07 (2.63)	9.03 (2.88)	0.97	< 0.001	0.82	1.1
Total score on motivation, three items	10.64 (2.87)	10.81 (2.92)	0.17	< 0.05	0.04	0.30
Total score on risk reduction, three items	8.18 (2.43)	8.85 (2.67)	0.67	< 0.001	0.54	0.81
Score on acceptance for blood sampling, one item	2.85 (1.02)	3.09 (1.09)	0.24	< 0.001	0.18	0.29

Time 1: Before reading the brief summary

Time 2: After reading the brief summary

## Discussion

This research showed that social trust in medical students was associated with the perception of ability and the perception of value similarity, which were introduced using the SVS model. The citizens’ perception of value similarity and ability explained the trust and the perception of risk reduction in medical students. Furthermore, information on the medical school curriculum and student doctor certification significantly improved the social trust in medical students, especially regarding trust, ability, and value similarity. These findings support hypotheses 1 and 2 of this work.

Citizens’ perception of ability had the most significant effect on trust in medical students, followed by value similarity, as shown in Fig. 4. The path coefficient of ability on trust in medical students is likely more vital than the path coefficient ability on trust in groups involved in the Great East Japan Earthquake, as shown in a previous study [24]. Because medical care concerns life, death, and suffering, medical students are required to be highly trained as medical professionals. Therefore, medical students may have to be perceived as being competent to be trusted by citizens. A systematic review of trust in medicine also pointed out the perspective of the physicians’ ability [15].

The perception of value similarity had the second most significant effect on social trust in medical students. The findings of this study of the impact of value similarity on trust also support previous research on the relationship between physician empathy and trust [12, 18, 19]. Patients seem to be concerned about the physicians’ intentions to act in their interest when trust in a particular physician has not been established [12]. The study participants may not have been intensely interested in, and have an unclear consensus regarding the practice of medical students, because most of them did not have the experiences of regular visits to hospitals and clinics and engagement with medical students, as shown in Table 1. In this context, the effect of value similarity may be diminished [23]. This result suggests the importance of focusing on the value similarity in clinical education, such as shared decisions, patient-centered medicine, and professionalism.

Informing citizens about the curricula of medical schools and the certification of medical students is important from both the perspective of research of social trust in physicians and decision making by the patients. From the viewpoint of social trust, this was the first report that intervened on social trust in medical students, including the estimation by the participants of the ability and value similarity of medical students. In this research, social trust in medical students as well as citizens’ perception of ability and value similarity and their acceptance for blood sampling were significantly improved after reading the brief summary, as shown in Table 4. There is insufficient evidence that a particular intervention improves the interpersonal trust of patients in their physician [4, 5, 15, 18, 32], in agreement with the results of social science research [20, 33], with the exception that the voluntary action of professionals showing value similarity can improve trust [20]. The brief summary of this research provided an explanation to citizens about the curricula of medical schools and the certification of medical students. This suggests the importance of informing citizens for their acceptance of medical practice by medical students in the clinical setting.

From the viewpoint of patient decision making, information about the certification of medical students and the education of knowledge, skills, and professionalism in medical schools is also helpful in improving social trust in medical students in clinical settings. The difficulty in improving social trust seems to be caused to the absence of access to information about the abilities and values of medical students. Estimating these abilities may be challenging for citizens, as reported for licensed physicians [12]. Moreover, medical students do not have a physician’s license, and physician certification is fundamental for trust [34]. In this context, citizens are forced to over-adapt the information available for establishing trust or distrust in medical students. Trust has the function of decreasing complexity by over-adapting the information [35]. Before reading the brief summary about medical students, citizens would use knowledge obtained from mass communication outlets, such as television [36, 37]. When citizens access correct information about the certification of medical students and the curricula of medical schools, they may correctly decide to trust medical students or not based on the estimation of their ability and value similarity.

In the current research, risk reduction had a different position in the MS-ST model compared with the SVS model reported in previous research [20, 24]. This change was statistically developed by model fit comparisons using SEM, as shown in Table 2. In addition to the statistical examination, the results appear valid from the following aspects. Risk reduction is the evaluation of others, just like trust. Therefore, risk reduction and trust can be placed at the second level of the model. Conversely, the acceptance for blood sampling is a citizen’s decision (whether or not to accept a medical procedure) and has a different feature from the other five factors. It is theoretically possible that the behaviors of citizens seem to be impacted by their perceptions of medical students. Therefore, setting the acceptance for blood sampling to the third level of the model would be logically accepted. Considering that the achievements and failures of trainees in the clinical environment are regarded as the outcomes of the actions of their supervisors by the patients [38], risk reduction in medical students seems to include social trust and the perception of risk reduction in their supervisors. Additionally, the participants may assume that medical students perform medical procedures under supervision. To refine the MS-ST model of this study, further analyses are required to explain social trust in medical students and supervisors. Furthermore, trust in medical students should be studied among patients and their families, who are familiar with the medical-student–supervisor complex.

This study also showed that improving the perception of citizens of the ability and value similarity of medical students enhanced the acceptance for blood sampling by medical students through social trust and the perception of risk reduction in medical students. This finding is understandable, as allowing medical practice consists in risk perception based on trust in medical professions [4]. This study statistically clarified the impact of the information provided by the medical education system and the certifications on social trust in medical students. Practical implications for medical education and social psychological research have been shown by this study. The relationship between trust and medical procedures other than blood sampling is out of the scope of this article and needs to be further explored.

Increasing social trust, which is the basis of interpersonal trust [6, 9, 10], would enable medical students to become more trustworthy in an actual clinical setting. We may adapt this result to the recruitment of patients and families, to facilitate the administration of medical procedures by medical students for medical education. As shown here, the social trust model in medical students will aid medical schools explain why medical students are trustworthy to citizens.

### Limitations

Our research had several limitations. First, no causality could be established because this study had a cross-sectional design. However, we addressed the weakening of the vulnerability by engaging in pre-and post-questionnaire surveys using MGA.

Second, because the study was conducted in Japan, which has an Eastern cultural background, additional research is required for extrapolation of the results to regions with different cultural backgrounds, such as Western countries. Cultural differences regarding autonomy [39] may make a difference in the impact of value similarity on trust, although a previous study exploring the trust of patients in physicians in Japan indicated the presence of similarities between Japan and the U.S [10]. The effect of value similarity on trust in the Western culture is potentially greater than the impact of value similarity on trust demonstrated in this study in an Asian culture setting, because the autonomy of patients is lower in Japanese compared with U.S. culture [40].

Third, the variables used to measure concepts such as trust are self-descriptive. The fact that this research was based on a self-descriptive survey does not hinder its aims, which consisted in the exploration of how citizens subjectively trust medical students, the evaluation of their value similarity and ability, and the acceptance of procedures performed by medical students.

Fourth, the web survey design might have affected the validity of responses since all questions had to be answered.

In this study, the effects of personal contact with medical students were not captured. However, the questionnaire texts were designed to minimize the impact on trust from personal contact with medical students. The sub-analysis demonstrated the influence of the experience of medical practice with medical students in Additional file 4. Trust in medical students and the acceptance for blood sampling showed no significant differences with or without previous experience in medical practice by medical students. Given both points, the influence of personal contact with medical students on the social trust in medical students explored in this study is expected to be minor.

## Conclusions

We demonstrated that the perception of ability and value similarity introduced using the SVS model was associated with social trust in medical students in pre-graduate medical education in an Asian culture context. We also showed that facilitating information on the medical student certification system and the curriculum of pre-graduate medical education might improve social trust in medical students in Japan. To the best of our knowledge, the present study provided the first evidence of the relationship between social trust in medical students, the perception of ability, and value similarity. Further research is needed to explore the impact of the social trust in the supervisors of medical students. Moreover, the examination of the effect of social trust in medical students on the actual actions of citizens in a clinical environment is required.

## Electronic supplementary material

Below is the link to the electronic supplementary material.

Social trust questionnaire: Trust questionnaire used for evaluating social trust in medical students (Japanese version).

Brief summary used in this study: Brief summary of the medical education system and certification of medical students in Japan.

The SVS model initially assumed and the modified model: The SVS model initially assumed and the modified model.

The comparison according to medical practice experience with medical students: The sub-analysis to explore the influence of medical practice experience with medical students.

## Data Availability

The datasets used and analyzed during the current study are available from the corresponding author on reasonable request. The dataset supporting the conclusions of this article is securely stored at the University of Tokyo.

## Abbreviations

SVS: Salient Value Similarity
CFA: Confirmatory Factor Analysis
MGA: Multiple Group Analysis
SEM: Structural Equation Modeling
CFI: Comparative Fit Index
SRMR: Standardized Root Mean Square Residual
RMSEA: Root Mean Square Error of Approximation
