# Supplementary material for: Impact of value similarity on social trust in medical students: a cross-sectional web survey
Source: BMC Med Educ. 2023 Jul 24;23:528. doi: 10.1186/s12909-023-04493-w (PMC10367362; doi:10.1186/s12909-023-04493-w)
Supplement: Supplementary file 1 — Social trust questionnaire: Trust questionnaire used for evaluating social trust in medical students (Japanese version). [file 12909_2023_4493_MOESM1_ESM.docx]

**Additional file 1** Social trust questionnaire

Please answer the following questions if you were a patient at a hospital or clinic and received medical treatment, such as a medical interview, medical examination, or blood sampling.

Medical students:

1. Can be trusted.

2. Can be relied upon.

3. Can be entrusted.

4. Share the same point of view as mine.

5. Understand my feelings.

6. Priorities match mine.

7. Have high expertise.

8. Are efficient.

9. Have abundant expert knowledge.

10. Try their best.

11. Work very hard.

12. Are passionate

13. Can help ensure patient safety.

14. Can eliminate danger to patients.

15. Contribute to the prevention of medical errors.

16. I can accept blood sampling by medical students.

The questionnaire was used in this study to evaluate social trust and value similarity, ability, risk reduction, and the acceptance for blood sampling, which were hypothesized to correlate with social trust. No. 1–3 measure trust, No. 4–6 measure value similarity, No. 7–9 measure Ability, No. 10–12 measure motivation, No. 13–15 measure risk reduction, and No. 16 measure acceptance for blood sampling.
