# Supplementary material for: Impact of value similarity on social trust in medical students: a cross-sectional web survey
Source: BMC Med Educ. 2023 Jul 24;23:528. doi: 10.1186/s12909-023-04493-w (PMC10367362; doi:10.1186/s12909-023-04493-w)
Supplement: Supplementary file 2 — Brief summary used in this study: Brief summary of the medical education system and certification of medical students in Japan. [file 12909_2023_4493_MOESM2_ESM.docx]

**Additional file 2** Brief summary used in this study.

Before they can engage in medical practice, medical students are required to spend 4 years after entering university learning about the structure of the human body, nature of diseases and illnesses, and ethics expected of physicians. They learn through classroom learning as well as actual medical procedures such as medical interviews, examinations, and blood sampling via simulators and simulated patients.

In addition, students must demonstrate their ability to perform such medical procedures safely by passing the nationwide common examination for certification as a Student Doctor. The examination is taken by all medical students in Japan and consists of two tests: a computerized test of medical knowledge and a practical examination, such as a medical interview and blood sampling.

Medical students who successfully pass the two tests and fulfill the advancement requirements of their respective universities are accredited as Student Doctors by the Association of Japan Medical Colleges. The updated Medical Practitioners’ Act allows medical students certified as Student Doctors to perform medical procedures in hospitals to acquire the knowledge and skills required of a physician in clinical training. An example of medical practice undertaken by such medical students certified as Student Doctors is the influenza vaccination of nurses and doctors at the University of Tokyo Hospital.
