# Supplementary material for: Impact of value similarity on social trust in medical students: a cross-sectional web survey
Source: BMC Med Educ. 2023 Jul 24;23:528. doi: 10.1186/s12909-023-04493-w (PMC10367362; doi:10.1186/s12909-023-04493-w)
Supplement: Supplementary file 3 — The SVS model initially assumed and the modified model: The SVS model initially assumed and the modified model. [file 12909_2023_4493_MOESM3_ESM.docx]

**Additional file 3 The SVS model initially assumed and the modified model.**

(a) SVS model initially assumed


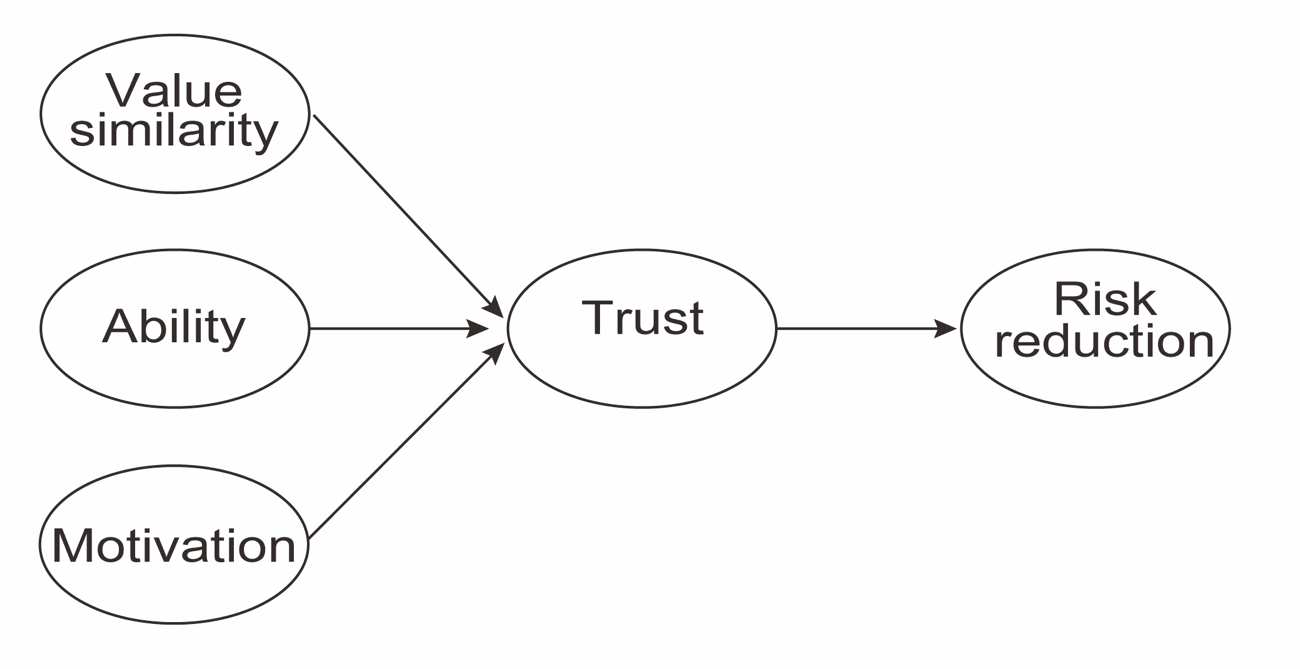


(b) The modified model


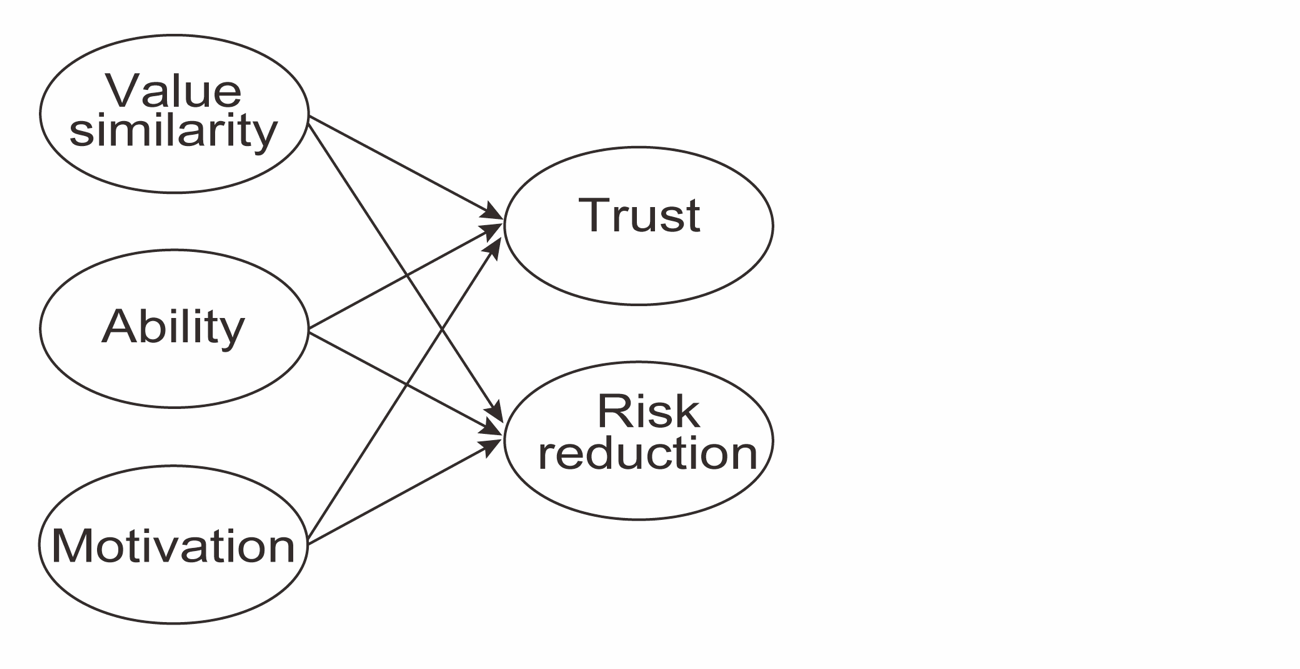


(a) The SVS model initially assumed, along with previous studies [7, 20, 24].

(b) The modified model accepted in this study.
