# Supplementary material for: Impact of value similarity on social trust in medical students: a cross-sectional web survey
Source: BMC Med Educ. 2023 Jul 24;23:528. doi: 10.1186/s12909-023-04493-w (PMC10367362; doi:10.1186/s12909-023-04493-w)
Supplement: Supplementary file 4 — The comparison according to medical practice experience with medical students: The sub-analysis to explore the influence of medical practice experience with medical students. [file 12909_2023_4493_MOESM4_ESM.docx]

**Additional file 4** The comparison according to the medical practice experience with medical students.

**Additional file 4-A** The comparison according to the medical practice experience with medical students before reading the brief summary.

| Experience of medical practice  by medical students | Experienced  N = 182 | Not experienced  N = 476 | *P*-value |
| --- | --- | --- | --- |
| Total score on trust,  three items* | 7.51 (2.73) | 7.52 (2.35) | 0.950 |
| Total score on value similarity,  three items* | 7.75 (2.65) | 7.94 (2.49) | 0.400 |
| Total score on ability,  three items* | 7.93 (2.99) | 8.12 (2.48) | 0.423 |
| Total score on motivation,  three items* | 10.69 (3.09) | 10.62 (2.78) | 0.802 |
| Total score on risk reduction,  three items* | 8.07 (2.66) | 8.23 (2.34) | 0.448 |
| Score on acceptance for blood sampling,  one item* | 2.79 (1.12) | 2.87 (0.98) | 0.334 |

* mean (SD)

The sub-analysis results to explore the influence of medical practice experience with medical students on social trust in medical students.

**Additional file 4-B** The comparison according to the medical practice experience with medical students after reading the brief summary.

| Experience of medical practice  by medical students | Experienced  N = 182 | Not experienced  N = 476 | *P*-value |
| --- | --- | --- | --- |
| Total score on trust,  three items* | 8.49 (3.09) | 8.69 (2.63) | 0.409 |
| Total score on value similarity,  three items* | 8.50 (2.93) | 8.67 (2.55) | 0.463 |
| Total score on ability,  three items* | 9.01 (3.20) | 9.04 (2.76) | 0.878 |
| Total score on motivation,  three items* | 10.74 (3.11) | 10.84 (2.85) | 0.689 |
| Total score on risk reduction,  three items* | 8.69 (3.02) | 8.92 (2.52) | 0.337 |
| Score on acceptance for blood sampling,  one item* | 3.03 (1.22) | 3.11 (1.04) | 0.450 |

* mean (SD)
